# Medical Clinic: Diseases of the Chest

**Published:** 1843-07-22

**Authors:** 


2. *Medical Clinic: Diseases of the Chest.* By G. Andral. *Condensed and Translated, with Observations.* By D. Spillan, M. D., &c. &c. Philadelphia: Edward Barrington & George D. Haswell; 1843. 8vo. pp. 408.

The classical work of Dr. Andral is too well known to need any encomium from us; our object in this place is simply to announce the republication of the English translation of Dr. Spillan. The work is now complete, and is placed within the reach of all physicians and students; and with none can they familiarize themselves with greater certainty of profit than the "Clinique Medicale." The cases are, in general, admirably reported, and in a style at happy variance with the one now the vogue in Paris. The perplexing amplification of the latter, with its wearisome repetitions and trite details, contrasts with the nervous and vigorous sketches of Dr. Andral, which present with felicitous consciseness, at a *coup d'œil,* all the important data of the case, and leave them impressed on the mind. The remarks, too, are the offspring of a philosophical as well as practical mind. We are never startled by the announcement of any absurdity, revolting to common sense, and opposed to all experience, for the sake of degrading the human mind to the capacity of a calculating machine.

Dr. Andral's advance to reputation has been as steady as it is assured. His position is not due to the uncertain and fitful enthusiasm of a few ardent young men, captivated by impracticable novelties. His fame is grounded on his known and tried abilities, his habits of industry and research, the fine philosophical cast of his mind, elucidating and *organizing* the difficulties of our science, and above all, for the truth and honesty of his character. And all this, too, is united to an unaffected diffidence, and a modesty of manner, as visible in the lecture room, where he is surrounded by one of the largest classes of the medical metropolis, as in productions of the closet.

